# Transcriptional profiling of PBMCs unravels B cell mediated immunopathogenic imprints of HCV vasculitis

**DOI:** 10.1371/journal.pone.0188314

**Published:** 2017-12-11

**Authors:** Emily Comstock, Cheol-Woo Kim, Alison Murphy, Benjamin Emmanuel, Xi Zhang, Michael Sneller, Bhawna Poonia, Shyamasundaran Kottilil

**Affiliations:** 1 Division of Clinical Care and Research, Institute of Human Virology, University of Maryland School of Medicine, Baltimore, MD, United States of America; 2 Department of Internal Medicine, Inha University, Incheon, South Korea; 3 Laboratory of Immunoregulation, National Institute of Allergy and Infectious Diseases, National Institutes of Health, Department of Health and Human Services, Bethesda, MD, United States of America; Saint Louis University, UNITED STATES

## Abstract

B cell depletion therapy using rituximab has been shown to be effective in achieving remission in patients with HCV-mixed cryoglobulinemic (MC) vasculitis. Previously, we have demonstrated abnormalities in peripheral immune cells involving neutrophils, chemotaxis, and innate immune activation among patients with HCV-MC vasculitis when compared to HCV patients without vasculitis. In this study, we evaluated the effect of B cell depletion therapy on transcriptional profiles of peripheral blood mononuclear cells before and after riruximab therapy, in order to unravel the pathogenic mechanism involved in HCV-MC vasculitis induced by abnormal B cell proliferation. DNA microarray analysis was performed using RNA from PBMCs from seven patients with HCV-MC vasculitis and seven normal volunteers. DNA was hybridized to Affymetrix U133A chips. After normalization, differentially expressed gene list with treatment was generated using partitional clustering. RT-PCR, flow cytometry, and enzyme immunoassay (EIA) was used to validate DNA microarray findings. Differentially expressed genes included B cells and non-B cell genes. Validation of genes using purified cell subsets demonstrated distinct effect of B cell depletion therapy on non-B cells, such as monocytes, T cells, and NK cells. Notably, B lymphocyte stimulator (BLyS) levels were persistently elevated in patients who subsequently relapsed. In conclusion, pathogenesis of HCV-MC vasculitis is mediated by abnormal proliferation of B cells, driven by BLyS, leading to significant effects on non-B cells in mediating symptomatology. Future therapeutics using a combination approach of B cell depletion and proliferation may be desired to achieve long-term remission.

## Introduction

While estimates vary, chronic hepatitis C (CHC) infection is present in approximately 71 to 170 million people globally [1–2]. Hepatitis C virus (HCV) is a single-stranded RNA Flavivirus that preferentially infects human hepatocytes [3]. Over time, CHC can lead to progressive liver fibrosis and cirrhosis of the liver. CHC is also the leading cause of hepatocellular carcinoma and liver transplantation [4–5]. A unique feature of CHC is the association with several extrahepatic manifestations, among which most commonly include: mixed cyroglobulinemic (MC) vasculitis, lymphoproliferative disorders, and insulin resistance [6–7]. Of these, Type II MC vasculitis is the most strongly associated with, and directly attributed to, CHC as more than 80% of patients with persistent MC vasculitis are seropositive for HCV [8–10]. Additionally, MC vasculitis is known to be a negative prognostic factor of virological response to HCV treatment and is generally associated with a high morbitity and mortality rate [11–12].

The pathogenesis of HCV-associated MC vasculitis is characterized by a preferential expansion of B cells, which are presumably triggered by HCV antigens or epitopes [8, 13–14]. These clonally expansive B cells produce soluble IgM with rheumatoid factor activity that has been shown to develop into immune complexes [15]. These complexes subsequently deposit in small vessels, ultimately resulting in vasculitis [8, 13]. The disease manifests with tissue and organ damage, particularly of the kidneys (glomeruli) and the skin. As a result, common clinical manifestations include membranoproliferative glomerulonephritis and cutaneous vasculitis [6, 16–17].

Various studies have demonstrated that patients diagnosed with MC vasculitis can be effectively treated with B cell depletion therapy [17–23]. B lymphocyte stimulator (BLyS, also known as the B cell–activating factor belonging to the TNF family, or BAFF) plays a major role in B cell homeostasis [24]. The BLyS protein is expressed as a trimer on monocytes, activated neutrophils, T cells, and dendritic cells [25–27], but can also be released into the circulation. Leading to the scertion of inflammatory cytokines, such as IL-2, TNF-α, and IFN-γ [26, 28–29]. BLyS can bind to 3 receptors: BLyS receptor 3 (BR3; also known as BAFF-R), transmembrane activator–1 and calcium modulator and cyclophilin ligand–interactor (TACI), and B cell maturation antigen (BCMA). BLyS is the sole ligand for BR3, whereas TACI and BCMA each can bind either BLyS or another TNF family ligand known as a proliferation-inducing ligand (APRIL) [30]. These ligand-receptor interactions vary in affinity: BLyS binds more strongly to BR3 than to TACI or BCMA, whereas APRIL displays the reverse affinity hierarchy. Elevated serum BLyS levels are frequently observed in patients with autoimmune Systemic lupus erytematosus (SLE). The use of a fully human monoclonal antibody that binds soluble BLyS (i.e., belimumab) in serologically active SLE patients has resulted in reductions in disease activity and B cell populations, resulting in symptomatic relief for most patients [31]. However, these effects are not indefinitely sustained, as many patients experience rapid symptom relapse after repletion of B cells [19].

The pathogenesis of immunological abnormalities associated with MC are not entirely clear. It is not entirely understood how CHC can cause MC including whether preferential B cell expansion directly or indirectly contributes to the pathogenesis of MC [17]. The purpose of this study is to evaluate the transcriptional profiles of peripheral blood mononuclear cells, before and after B cell depletion therapy, to unravel the pathogenic mechanisms involved in both active MC vasculitis and relapse. In this study, we performed gene expression profile analysis of PBMCs from patients undergoing B cell depletion therapy before and after rituximab treatment. To determine the effect of B cell depletion on cell-specific transcriptional profile, a comparison group of healthy controls was included to understand the direct and indirect mechanisms involved in the pathogenesis of HCV vasculitis and relapse.

## Materials and methods

### Study subjects

PBMCs were isolated by venipuncture from healthy controls (N = 7) and HCV-MC vasculitis subjects (N = 7) both before and after rituximab treatment (Table 1). Controls were volunteers selected through the blood bank and were seronegative for HIV, HBV, and HCV. The HCV-MC vasculitis subjects were selected from an open-label, randomized controlled trial conducted at the National Institute of Allergy and Infectious Diseases at the National Institutes of Health [17, 20]. Inclusion criteria mandated that subjects have active manifestations of MC vasculitis. In addition, these patients must not have responded to interferon-alpha and ribavirin. Of note, as described in Sneller et al., these treatments were indicated as first-line therapy for mild to moderate HCV-MC vasculitis at the time of the study [17, 20, 32]. Rituximab (375 mg/m^2^ per week for 4 weeks) was given to the 7 HCV-MC vasculitis subjects. The primary endpoint was remission at 6 months from study entry. All donors signed informed consents approved by the National Institute of Allergy and Infectious Diseases Institutional Review Board. We used clinical protocols NCT00029107, NCT00001281, and NCT00076427 to enroll study subjects [17]. This study was specifically approved by the above named institutional review board.

**Table 1:** Demographics and clinical characteristics of study participants.

Group	Age	Gender	Race	Risk	HCV GT	HCV VL	HIV Ab	HIV VL	Systemic Corticosteroids	Clinical Manifestations of MC Vasc
NV 1	41	F	White	N/A	N/A	N/A	Neg.	N/A		
NV 2	34	M	White	N/A	N/A	N/A	Neg.	N/A		
NV 3	56	F	White	N/A	N/A	N/A	Neg.	N/A		
NV 4	37	F	Black	N/A	N/A	N/A	Neg.	N/A		
NV 5	42	M	White	N/A	N/A	N/A	Neg.	N/A		
NV 6	46	F	White	N/A	N/A	N/A	Neg.	N/A		
NV 7	39	M	Hispanic	N/A	N/A	N/A	Neg.	N/A		
HCV MC Vasc 1	56	M	White	Needle Stick	1a	3,419,770	Neg.	N/A		Arthralgia, purpura, peripheral neuropathy
HCV MC Vasc 2	52	M	White	Transfusion Acquired	1a, 1b	1,864,910	Neg.	N/A	Prednisone 10 mg daily	Arthritis, purpura, peripheral neuropathy
HCV MC Vasc 3	47	F	White	IVDU	2b	2,296,250	Neg.	N/A		Peripheral neuropathy, hematuria
HCV MC Vasc 4	56	M	White	Intranasal Cocaine	1a	50,816	Neg.	N/A		Purpura, glomerulonephritis
HCV MC Vasc 5	47	M	White	Intranasal Cocaine	1a	932,880	Neg.	N/A	Prednisone 50 mg daily	Purpura, ulcers, mononeuritis
HCV MC Vasc 6	56	M	White	IVDU	1a	100,907	Neg.	N/A		Arthralgia, purpura, peripheral neuropathy
HCV MC Vasc 7	58	F	White	IVDU	1	<615	Neg.	N/A	Prednisone 30 mg daily	Purpura, ulcers, hematuria

### Isolation of PBMCs and RNA

We isolated PBMCs from white blood cells by the standard Ficoll-Hypaque Plus (Amersham Biosciences, Uppsala, Sweden) density gradient separation technique; they were then frozen for storage [17]. RNA was isolated following the manufacturer’s protocol, for the purpose of DNA microarray and qRT-PCR analysis using the Qiagen mRNA isolation kit (Qiagen, Germantown, MD) [17].

### DNA microarray analysis

Complementary DNA was prepared from total RNA and hybridized to Affymetrix U133A 2.0 oligonucleotide arrays according to the previously described manufacturer’s protocols (Affymetrix, Santa Clara, CA) [17, 33]. A significant analysis of microarray (SAM) algorithm was used to determine the genes that were differentially expressed after an extensive filtering process [17]. We eliminated genes with low variability or undetectable expression levels (for the majority of samples) from analysis if the Guanosine-Cytosine Robust Multi Array values for these genes were within the interquartile range of <0.263 or a 75^th^ percentile of <5 [17]. The corresponding genes and samples from the individuals were then subjected to partitional clustering [17].

### Real-time quantitative reverse transcription polymerase chain reaction (qRT-PCR)

As previously described, total RNA that was isolated from PBMCs was reverse-transcribed using random primers with the High Capacity cDNA Reverse Transcriptase Kit (Life Technologies) [17]. Between 1 and 25 ng of RNA was used for each qRT-PCR reaction. Except where indicated, Taqman expression assays were run with technical duplicates (Life Technologies). Primer/probe sets were purchased from Life Technologies and were pre-designed for respective genes. Gene expression was determined as a cycle at threshold (Ct) based on 40 PCR cycles. For statistical analysis, undetectable expression was assigned a minimal detectable level with a Ct value of 40. Expression of *GAPDH* was used as an endogenous control, with *GAPDH* Ct values for all samples being distributed between 20 and 25. Relative expression of targets was calculated as dCt values (normalized by *GAPDH* Ct values) or ddCt values (to calculate fold change compared to other samples). Expression reactions were run in 96-well plates on a 7500 Real-Time PCR System (Applied Biosystems).

### Measurement of BLyS levels

Fresh serum was collected from patients before and after rituximab therapy. These serum samples were stored at -80C until further use. At the time of the assay, serum was thawed and aliquoted to use in an EIA to detect levels of BLyS as previously described [33]. Soluble BLyS was expressed as ng/ml.

### Phenotypic analysis of B Cells

Multicolor flow cytometry analyses were performed on whole blood to determine lymphocyte counts and the frequency of each B-cell subpopulation. Lymphocyte counts were performed by a core NIAID facility following standard procedures. The following fluorochrome-conjugated monoclonal antibodies were used to stain B cells: allophycocyanin (APC) anti-CD10, APC-H7 anti-CD20, fluorescein isothiocyanate (FITC) anti-IgM, and phycoerythrin (PE) anti-IgG (BD Biosciences), PerCP-Cy5.5 anti-CD19 and PE-Cy7 anti-CD27 (eBioscience), FITC anti-CD21 (Beckman Coulter), and FITC anti-IgA (Dako). Analyses were performed on a Canto flow cytometer (BD Biosciences) with FlowJo Version 8.6 software (TreeStar) as previously described [34].

### Statistical analysis

A paired *t*-test was used to compare the difference in the paired responses for patients between pre-therapy and post-therapy. A Wilcoxon-Mann-Whitney test was used to compare the difference in the relative geneexpression in the two independent groups between the control group and rituximab therapy group (either at pre-therapy or post-therapy). All analysis was conducted in GraphPad Prism 6.0 with a *P*-value < 0.05 indicating statistical significance.

## Results

### Changes in peripheral B cell phenotype associated with HCV-MC vasculitis

Since abnormal proliferation of B cells is the pathognomonic feature of HCV-MC vasculitis, we sought to investigate whether the distribution of B cells were also affected by HCV-MC vasculitis. In this regard, we performed phenotypic analysis of peripheral B cells in patients with HCV-MC vasculitis and healthy controls, as previously described. B cell phenotype in patients with HCV-MC vasculitis was distinct from healthy controls. Specifically, there was an increase in CD21 and tissue-like memory B cells as well as decreased naïve and memory B cells as compared to healthy controls (S1 Fig). These results suggest a persistent abnormal B cell phenotype in HCV-MC vasculitis patients, which is indicative of ongoing B cell proliferation and exhaustion.

### Differential gene expression profiles in PBMCs of HCV-MC vasculitis subjects before and after B cell depletion therapy

We performed DNA microarray analysis to compare the host gene expression profile induced by B cell depletion therapy in PBMC from HCV- MC vasculitis patients. In order to perform this analysis, we used RNA isolated from PBMCs from patients with HCV-MC before and after B cell depletion therapy with rituximab. PBMCs from healthy controls were used as controls.

**Fig 1:**
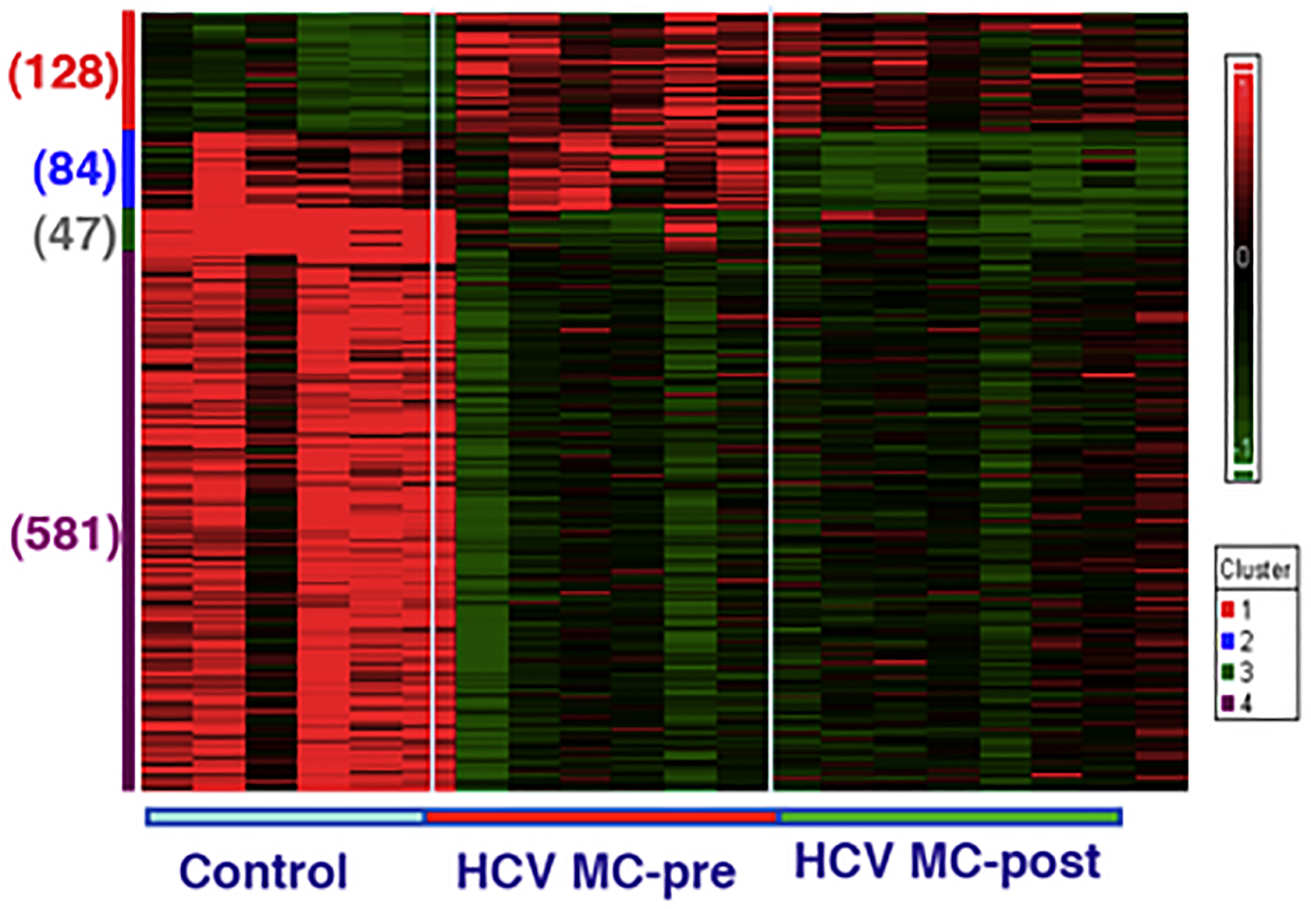
Clustering of differentially expressed genes in PBMCs from two patient cohorts. Levels of gene expression were assayed using Affymetrix Human Genome U133A chips as described in the methods section. A total of 529 differentially expressed genes were identified from 20 total samples for 7 patients during pre and post treatment. All samples were hybridized on Hu133plus 2.0. Genes were selected by filters: P value< = 0.05 and Fold Change 2 in any of the above 4 comparisons. Genes were grouped using *K*-means clustering and samples were grouped by hierarchical clustering. Differences in relative levels of gene expression (*Z*-score) are represented by color; red indicates up-regulation while green indicates down-regulation corresponding to gene expression in controls. The numbers in the parenthesis on the left signify the number of genes in each cluster. Cluster 1 represents genes down-regulated in HCV mono-infected patients and HCV MC vasculitis patients. Cluster 2 represents genes up-regulated in HCV mono-infected patients without vasculitis. Cluster 3 represents genes up-regulated in HCV MC vasculitis patients and Cluster 4 represents genes down regulated in HCV MC vasculitis patients.

Using Affymetrix human genome U133A oligonucleotide arrays and a SAM algorithm, we identified a total of 840 differentially expressed genes between the three groups (Fig 1). The corresponding genes and samples from the individuals were subjected to partitional clustering which revealed four distinct clusters of differential gene expression (Fig 1). Cluster 1 consists of 128 genes that are up-regulated in HCV-MC vasculitis patients before and after B cell depletion therapy. Functional annotation analysis revealed that these genes share roles in cellular defense. Cluster 2 includes 84 genes that are down-regulated in HCV-MC vasculitis subjects after rituximab. Cluster 2 genes are mostly associated with B cell phenotype and function. Cluster 3 consists of 47 genes that are up-regulated in HCV-MC vasculitis patients as compared to healthy controls. Cluster 4 includes 581 genes that are down-regulated in HCV-MC vasculitis patients.

Representative genes that belong to each cluster were identified by rigorous literature-mining algorithms, significance of microarray analysis, and biology of the disease processes of HCV-MC vasculitis and B cell depletion therapy (Table 2). This process of gene selection is consistent with our previous studies and was validated by qRT-PCR [33].

**Table 2:** List of biologically relevant genes identified by DNA microarray analysis and literature-driven algorithm.

**Cluster 1: Up-regulated in HCV-MC vasculitis patients before and after B cell depletion therapy**			
**Gene ID**	**Name**	**Short Name**	**Function**
433	Asialoglycoprotein receptor 2	Asgr2	Encodes a subunit of the asialoglycoprotein receptor which may facilitate hepatic infection
6347	Chemokine (C-C motif) ligand 2	CCL2	Displays chemotactic activity for monocytes and basophils only
6354	Chemokine (C-C motif) ligand 7	CCL7	Encodes monocyte chemotactic protein 3 which attracts macrophages during inflammation and metastasis
924	CD7 molecule	CD7	Encodes an immunoglobulin transmembrane protein which is essential in T-cell and T-cell/B-cell interactions
5610	Eukaryotic translation initiation factor 2-alpha kinase 2	EIF2AK2	Encodes a serine/threonine protein kinase, activated protein can inhibit protein synthesis
2867	Free fatty acid receptor 2	FFAR2	Encodes a GP40 G protein-coupled receptor involved in inflammatory response and lipid plasma levels regulation
3554	Interleukin 1 receptor, type I	IL1R1	A key mediator in cytokine-induced immune and inflammatory responses
5788	Protein tyrosine phosphatase, receptor type, C	PTPRC	Encodes protein tyrosine phosphatase, which regulates T and B cell antigen receptor signaling
7242	Tumor necrosis factor receptor superfamily, member 21	TNFRSF21	Activates nuclear factor kappa-B and mitogen-activated protein kinase 8 and induces cell apoptosis
**Cluster 2: Down-regulated in HCV-MC vasculitis subjects in RTX post treatment**			
**Gene ID**	**Name**	**Short Name**	**Function**
930	CD19 molecule	CD19	Encoded molecule decreases the threshold for antigen receptor-dependent stimulation of B lymphocytes
523	Interleukin-8 receptor, beta	CD182	Proinflammatory cytokine receptor
729230	Chemokine (C-C motif) receptor 2	CCR2	Encodes receptor which facilitates monocyte chemotaxis
10561	Interferon-induced protein 44	IFI44	Aggregates to form microtubular structures, associated with HCV infection
2537	Interferon, alpha-inducible protein 6	IFI6	Induced by interferon, involved in apoptosis regulation
3437	Interferon-induced protein with tetratricopeptide repeats 3	IFIT3	Inhibitor of cellular and viral processes, cell migration, proliferation, signaling, and viral replication
8638	2'-5'-oligoadenylate synthetase-like	OASL	Antiviral activity against encephalomyocarditis virus and HCV, binds double-stranded RNA
51330	Tumor necrosis factor receptor superfamily, member 12A	TNFRSF12a	Weak apoptosis inducer, promotes angiogenesis, proliferation of endothelial cells, cell adhesion to matrix proteins
**Cluster 3: Up-regulated in HCV-MC vasculitis subjects as compared to healthy controls**			
**Gene ID**	**Name**	**Short Name**	**Function**
3458	Interferon, gamma	IFNG	Encodes a cytokine with antiviral, immunoregulatory, and anti-tumor properties; potent macrophage activator
**Cluster 4: Down-regulated in HCV-MC vasculitis subjects as compared to health controls**			
**Gene ID**	**Name**	**Short Name**	**Function**
432	Asialoglycoprotein receptor 1	ASGR1	Encodes a asialoglycoprotein receptor, mediates the endocytosis and lysosomal activity of glycoproteins
9530	BCL2-associated athanogene 4	BAG4	Encodes BAG1, an anti-apoptotic protein
9560	Chemokine (C-C motif) ligand 4-like 2	CCL4L2	Encodes for cytokines that function in inflammatory and immunoregulatory processes
51744	CD244 molecule, natural killer cell receptor 2B4	CD244	Encodes receptor on NK and some T cells, regulates non-major histocompatibility complex restricted killing
948	CD36 molecule (thrombospondin receptor)	CD36	Encodes a platelet surface glycoprotein, a receptor for thrombospondin, functions in cell adhesion
115352	Fc receptor-like 3	FCRL3	Involved in immunoreceptor-tyrosine activation and inhibition, possible role in immune system regulation
3455	Interferon (alpha, beta and omega) receptor 2	IFNAR2	Forms one of the two chains of a receptor for alpha and beta interferons
5473	Pro-platelet basic protein (chemokine (C-X-C motif) ligand 7	PPBP	Encodes a platelet-derived growth factor that is a potent chemoattractant and activator of neutrophils
81793	Toll-like receptor 10	TLR10	Encoded protein is involved in pathogen recognition and activation of innate immunity responses
7100	Toll-like receptor 5	TLR5	Encoded protein is involved in pathogen recognition and activation of innate immunity responses
51284	Toll-like receptor 7	TLR7	Encoded protein is involved in pathogen recognition and activation of innate immunity responses
51311	Toll-like receptor 8	TLR8	Encoded protein is involved in pathogen recognition and activation of innate immunity responses

### Differential effect of rituximab on B cell and non-B cell gene expression

To validate our DNA microarray analysis, we performed qRT-PCR analysis on the most biologically relevant genes from each cluster selected as described above. Total RNA was extracted from the PBMCs of our same patient cohorts and subjected to qRT-PCR using primers specific for the validated genes.

**Fig 2:**
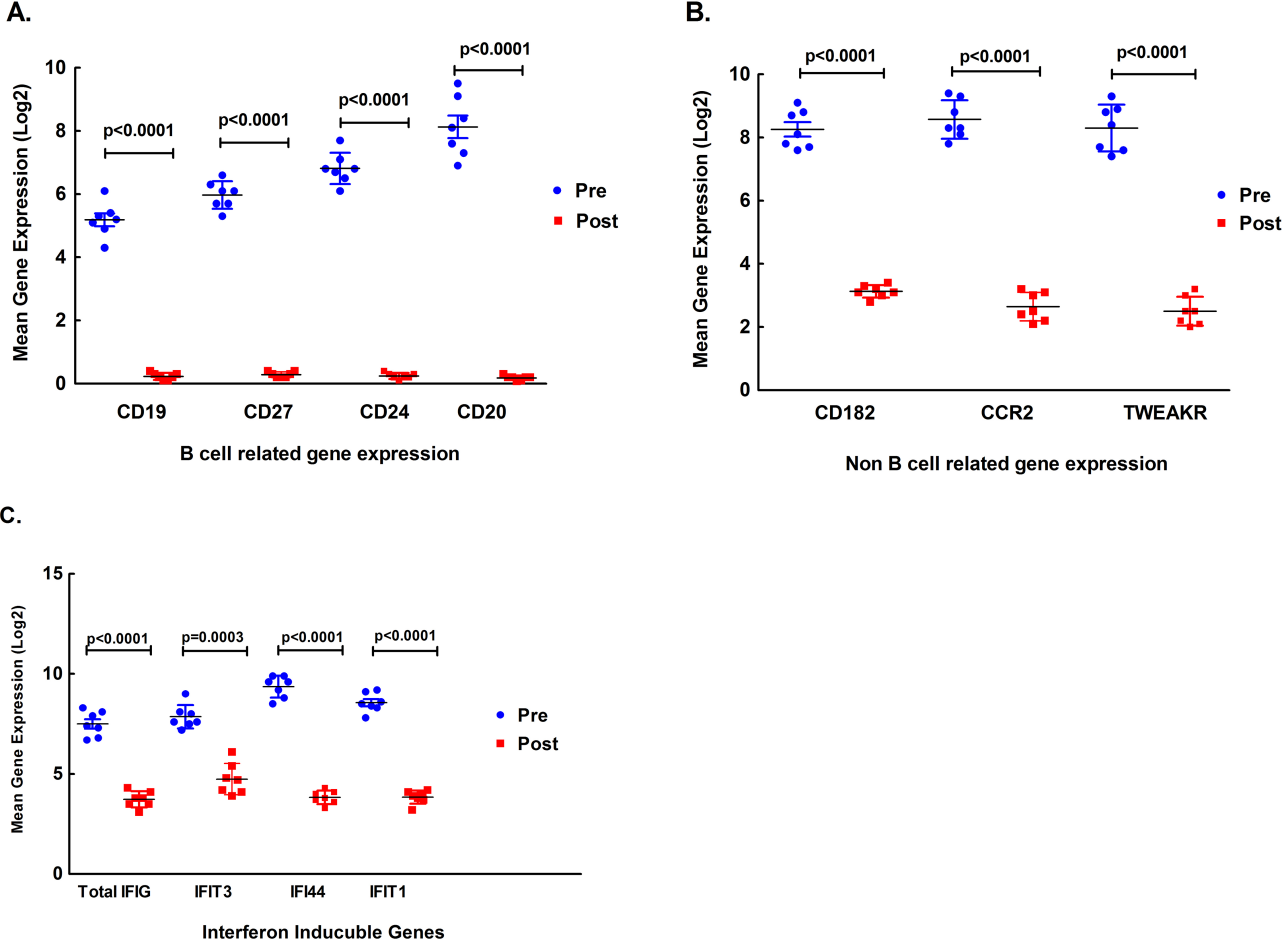
Validation of differentially expressed genes identified by DNA Microarray analysis. Expression of genes related and unrelated to B cells are down regulated with Rituximab (RTX) treatment. As shown in Fig A, expression of B cell related genes CD19, CD27, CD24, and CD20 were significantly lower after RTX treatment than at baseline (p<0.0001). Fig 2B shows expression of non-B cell genes CD182, CCR2, and TWEAKR were all down regulated after RTX treatment than at baseline (p<0.0001). Fig 2C shows expression of IFIGs including total IFIG (p<0.0001), IFIT3 (p = 0.0003), IFI44 (p<0.0001), IFIT1 (p<0.0001) that were down-regulated after RTX treatment.

As expected, all B cell related genes were down-regulated with rituximab treatment. As shown in Fig 2A, B cell genes such as CD19 (*P*<0.0001, means±standard deviation, 0.2±0.1), CD27 (*P*<0.0001, 0.3±0.1), CD24 (*P*<0.0001, 0.2±0.1), and CD20 (*P*<0.0001, 0.2±0.1) were significantly down regulated at the end of rituximab therapy, as compared to baseline expression levels. Surprisingly, we had several non-B cell related genes that were also down regulated with rituximab therapy. As shown in Fig 2B, the expression of CD182 (*P*<0.0001, 3.1±0.2), CCR2 (*P*<0.0001, 2..6±0.5), and TWEAKR (*P*<0.0001, 2.5±0.5) were expressed at lower levels at the end of rituximab therapy, as compared to baseline expression levels. These findings reflect the changes probably induced by the indirect effects of pathogenic B cell depletion in patients with HCV-MC vasculitis.

Previously, we have demonstrated that interferon inducible genes (ISG) were all up-regulated in PBMCs of patients with HCV-MC vasculitis [17]. In this study, we observed a significant down regulation of ISG expression at the end of rituximab therapy in patients with HCV-MC vasculitis.

### Evidence for BLyS in driving B cells contributing to the pathogenesis of HCV-MC vasculitis

**Fig 3:**
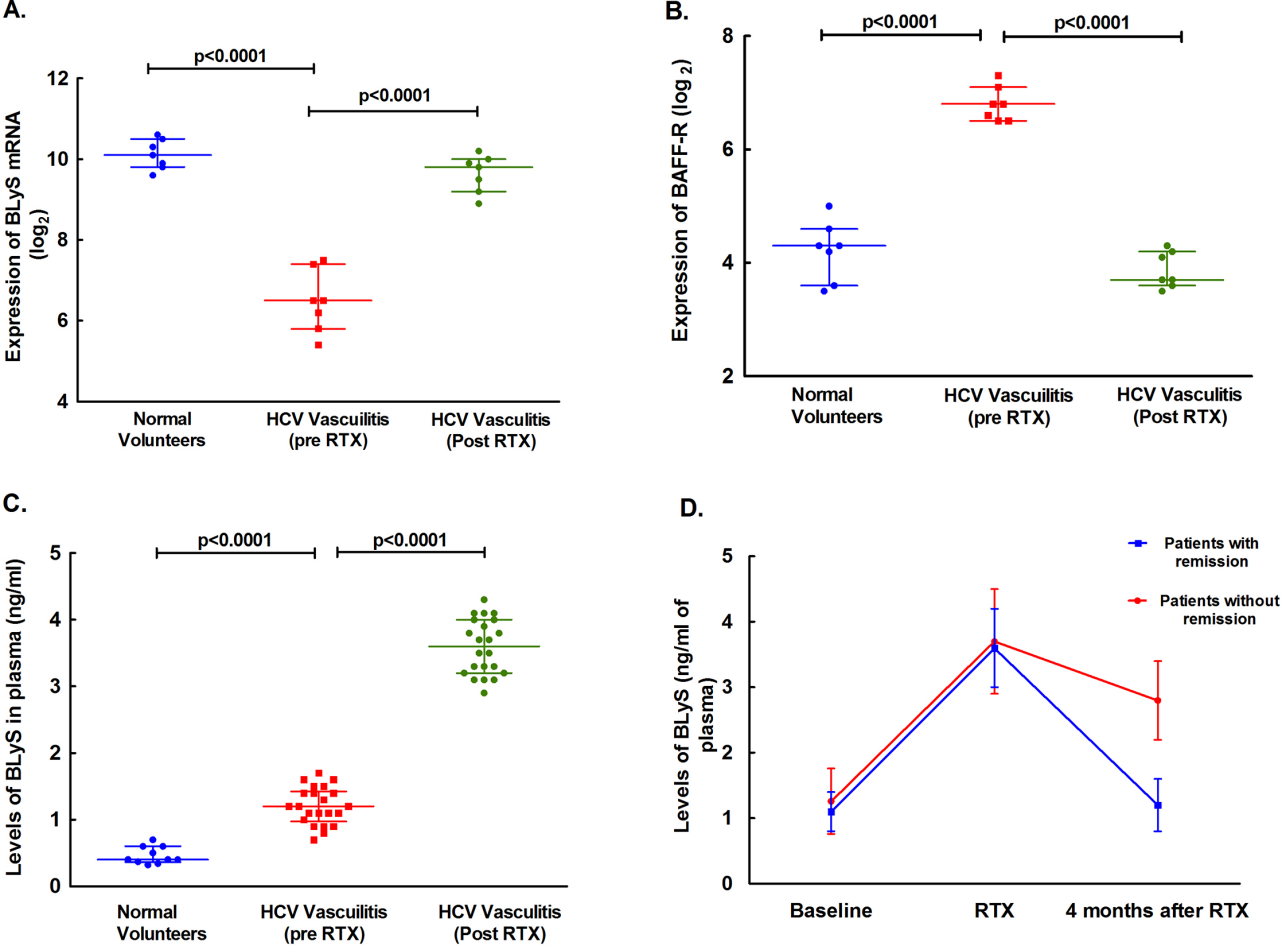
Regulation of BLyS and receptor expression of protein levels with Rituximab (RTX) treatment. As shown in 3A, expression of BLyS is down regulated in HCV vasculitis patients as compared to normal volunteers (p<0.0001), but is upregulated after RTX treatment (p<0.0001). BAFF-R expression is upregulated in HCV vasculitis patients as compared to normal volunteers (p<0.0001), but down regulated after RTX treatment (p<0.0001) in 3B. In 3C, levels of BLyS are upregulated in patients with HCV vasculitis as compared to normal volunteers (p<0.0001), but are even further upregulated after treatment with RTX (p<0.0001). In 3D, paradoxically BLyS levels remain elevated in relapsed patients who achieve remission 4 months after RTX therapy.

In order to understand B cell homeostasis after rituximab therapy, we evaluated gene expression profiles of non-B cell genes involved in B cell homeostasis. When we examined the levels of expression of receptors for BLyS (Fig 3A) and BAFF-R levels (Fig 3B) they were significantly lower than at baseline reciprocating the expression levels of BLyS (Fig 3C). In order to confirm BLyS mRNA levels, we quantified the levels of BLyS in serum by EIA assay. As shown in Fig 3C, the levels of BLyS were significantly higher in patients with HCV-MC vasculitis (*P*<0.05, 1.2±0.3 ng/mL) than in healthy controls, and and increased further after B cell depletion (p<0.05, 3.61±0.4 ng/mL). An interesting clinical observation is that many of the patients had a prolonged period of remission after B cell depletion therapy, while some experienced more rapid relapse [20]. All patients were followed at least monthly for a period of 12 months, exept for the rituximab group who were also seen weekly during the first month of treatment [20]. To investigate whether the elevated levels of BLyS contributed to relapse, we examined the relationship between elevated levels of BLyS and relapse, we found that patients who relapsed had a higher level of BLyS in serum, while those who remained in remission had declining levels of BLyS after rituximab therapy (Fig 3D; see Table 1 for patient demographics); a finding also previously reported for Sjögren's syndrome [35]. Overall, these results suggest a pathogenic role for BLyS in the persistence of abnormal B cell proliferative response in HCV-MC vasculitis.

## Discussion

Our study demonstrates key signatures associated with B cell pathogensesis of HCV mixed cryoglobulinemic vasculitis. In addition, our data show that elevated levels of BLyS is a likely factor that drives pathogenic B cell expansion in patiens with HCV-MC vasculitis and could be potentially used as a therapeutic target in refractory casess.

HCV-MC vasculitis, a common extrahepatic manifestation of CHC, has been shown to be a systemic small vessel vasculitis driven by monoclonal proliferation of B cells, subsequently producing IgM with rheumatoid factor activity [17, 20]. The pathogenesis of vasculitis includes complement mediated injury, particularly to the glomeruli and vascular endothelium, due to autoantibody, IgG, and HCV particle complex deposits. This in turn leads to skin ulcers, membranoproliferative glomerulonephritis, and possible end-organ damage [8–10, 17, 20]. Rituximab therapy has been shown to reduce symptoms by removing circulating B cells, resulting in prolonged remission for some and a more rapid relapse for others [19–23, 36–38]. In this regard, the cellular changes associated with B cell depletion have not yet been completely evaluated. We previously established that HCV-MC vasculitis patients also have other immune cells that are affected and contribute to the disease process [17]. In this regard, we had demonstrated a peripheral transcriptional profile that reflects innate immune activation and impaired monocyte chemotaxis and neutrophil function compared to CHC patients without vasculitis [17]. In this study, we further explored transcriptional profiling of peripheral lymphocytes before and after B cell depletion therapy. As expected, most genes down regulated after B cell depletion were B cell associated genes; this was confirmed by flow cytometry. We also examined the B cell phenotype in patients with HCV-MC vasculitis to identify abnormal B cell phenotypes that may be associated with the disease process. Clearly, B cell phenotypes were distinctly different from that seen with normal individuals, characterized by persistently elevated levels of tissue-like memory B cells and activated memory B cells. This is consistent with the chronic inflammatory process, which drives B cell activation [39].

Although many of the differently regulated genes were B cell specific, there were several pathways of gene expression that were affected by B cell depletion which were not associated with B cells. When we performed transcriptional analysis of genes in fractionated and total PBMCs, we were able to demonstrate the cell type specific downregulation of several pro-inflammatory genes such as CCR2, (monocytes, TWEAKR (T cells), CD79A (B cells), and interferon stimulated genes (monocytes)). This unique finding signifies the impact of the pathologic process underlying HCV-MC vasculitis and the role of B cells in driving inflammatory processes that result in the activation of other cells types. By performing a subtractional analysis (B cell depletion therapy) we were able to identify the effects of B cell proliferative disease on non-B cells.

Finally, the clinical data from the trial showed that rituximab therapy was highly effective in achieving remission on all treated patients [20]. However, the period of remission was highly variable between patients [20]. The exact nature of why some had an earlier relapse and others were able to sustain prolonged remission is unclear. Understanding of this process would be helpful in developing therapeutics aimed at prolonging clinical remission in HCV-MC vasculitis after B cell depletion.

Our results suggest evidence for non-B cell mediated effects in contributing to the pathogenesis of HCV-MC vasculitis. This finding is of particular interest in light of the positive effects of B cell depletion therapy, as previously observed in HCV-MC patients with severe liver disease and cirrhosis, whose pathogenesis is not traditionally B cell related [40–43]. In this regard, we investigated pathways of gene expression that were associated with driving B cell ontogeny and proliferation. Our results demonstrated a dichotomous effect of B cell depletion with the levels of BLyS in plasma. B cell depletion resulted in an increase in BLyS levels in plasma, which declined with recovery of B cells in most patients [44]. As one would expect, the expression of BAFF-R was inversely related to BLyS expression. To our surprise, when we examined the BLyS levels in plasma of patients who subsequently relapsed versus those who had long term remission, a strikingly significant correlation emerged. Patients who relapsed had persistently higher levels of BLyS in plasma compared to those who had long term remission. These findings suggest a pathogenic role for BLyS in driving an abnormal B cell response in patients with HCV-MC vasculitis [14, 45–50]. Furthermore, BLyS may be responsible for the relapse of symptoms after B cell deletion. BLyS is secreted by T cells and is a major cytokine responsible for maintaining B cell homeostasis. Abnormalities in BLyS secretion are related to other B cell associated pathologic conditions, such as systemic lupus eryhtematosis. Targeting BLyS using belimumab is now an FDA approved therapy for patients with Systemic Lupus Erythematosis [47]. Our data support a combination therapy using rituximab (B cell depletion) and belimumab (controlling B cell proliferation) in refractory HCV-MC patients ineligible to udergo HCV therapy [47].

Our study had a variety of strengths. In particular, we note the inclusion of a healthy control group (NV), both pre and post therapy clinical measurements, and sample collection for transcriptional profiling. Yet, our study was not without limitations. We recognize that the sample size is small. However, it is important to note that the disease is uncommon. HCV-MC vasculitis has an estimated prevelance of less than 1 percent in the nonselected HCV population in North America, where the study took place [51]. An additional weakness of this study is the short period of patient follow up, which was mandated by the original clinical trail design.

In conclusion, we are able to demonstrate B cell depletion therapy with rituximab results in both direct (on B cells) and indirect (non-B cells) effects on the host immune system. Direct effects on B cells include depletion of B cells and remission from HCV-MC vasculitis symptoms. We were also able to demonstrate indirect effects on most immune cell types including T cells, NK cells, and monocytes. Most importantly, we found that B cell depletion results in down regulation of interferon inducible genes in monocytes, which may have favorable implications for HCV therapy. We also unraveled a novel mechanism involving BLyS driving pathogenic B cells to result in clinical relapse after B cell depletion therapy. Therefore, a combination of B cell depletion and proliferation restarting therapy may be required for HCV-MC patients to maintain long term remission. Furthermore, we identified that persistent elevations in BLyS levels are associated with relapse of symptoms and could be a target for future therapeutics.

## Supporting information

S1 Fig Clustering of differentially expressed genes in PBMCs. Levels of gene expression were assayed using Affymetrix human genome U133A oligonucleotide arrays as described in the methods section. A total of 840 differentially expressed genes were identified. Genes were subjected to partitional clustering, revealing four distinct clusters of differential gene expression. Cluster 1 consists of 128 genes that are up-regulated in HCV-MC vasculitis patients before and after B cell depletion. Cluster 2 includes 84 genes that are down-regulated in HCV-MC vasculitis subjects after rituximab. Cluster 3 consists of 47 genes that are up-regulated in HCV-MC vasculitis patients as compared to normal volunteers. Cluster 4 includes 581 heat shock proteins that are down-regulated in HCV-MC vasculitis patients.
(TIFF)

S1 Table Differential Gene Expression Clusters 1–4. (XLSX)
