# Supplementary figures and images for: Transcriptional profiling of PBMCs unravels B cell mediated immunopathogenic imprints of HCV vasculitis
Source: PLoS One. 2017 Dec 11;12(12):e0188314. doi: 10.1371/journal.pone.0188314 (PMC5724854; doi:10.1371/journal.pone.0188314)

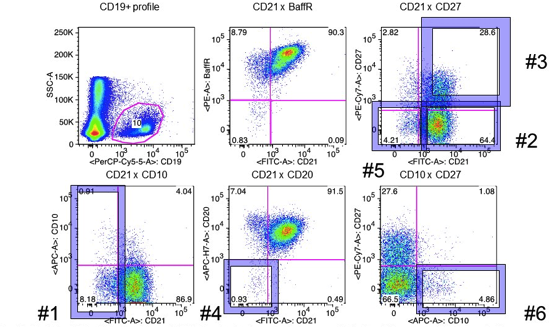

Supplement: S1 Fig — Levels of gene expression were assayed using Affymetrix human genome U133A oligonucleotide arrays as described in the methods section. A total of 840 differentially expressed genes were identified. Genes were subjected to partitional clustering, revealing four distinct clusters of differential gene expression. Cluster 1 consists of 128 genes that are up-regulated in HCV-MC vasculitis patients before and after B cell depletion. Cluster 2 includes 84 genes that are down-regulated in HCV-MC vasculitis subjects after rituximab. Cluster 3 consists of 47 genes that are up-regulated in HCV-MC vasculitis patients as compared to normal volunteers. Cluster 4 includes 581 heat shock proteins that are down-regulated in HCV-MC vasculitis patients. (TIFF) [file pone.0188314.s001.tiff]
